# Bromodomain inhibitor JQ1 reversibly blocks IFN-γ production

**DOI:** 10.1038/s41598-019-46516-x

**Published:** 2019-07-16

**Authors:** Hunter R. Gibbons, Deborah J. Mi, Virginia M. Farley, Tashawna Esmond, Mary B. Kaood, Thomas M. Aune

**Affiliations:** 1 Department of Pathology, Microbiology, and Immunology, Vanderbilt University, Nashville, TN 37232 USA; 2 Department of Medicine, Vanderbilt University Medical Center, Nashville, TN 37232 USA

**Keywords:** Immunology, Interferons

## Abstract

As a class, ‘BET’ inhibitors disrupt binding of bromodomain and extra-terminal motif (BET) proteins, BRD2, BRD3, BRD4 and BRDT, to acetylated histones preventing recruitment of RNA polymerase 2 to enhancers and promoters, especially super-enhancers, to inhibit gene transcription. As such, BET inhibitors may be useful therapeutics for treatment of cancer and inflammatory disease. For example, the small molecule BET inhibitor, JQ1, selectively represses *MYC*, an important oncogene regulated by a super-enhancer. IFN-γ, a critical cytokine for both innate and adaptive immune responses, is also regulated by a super-enhancer. Here, we show that JQ1 represses IFN-γ expression in TH1 polarized PBMC cultures, CD4+ memory T cells, and NK cells. JQ1 treatment does not reduce activating chromatin marks at the *IFNG* locus, but displaces RNA polymerase II from the locus. Further, IFN-γ expression recovers in polarized TH1 cultures following removal of JQ1. Our results show that JQ1 abrogates IFN-γ expression, but repression is reversible. Thus, BET inhibitors may disrupt the normal functions of the innate and adaptive immune response.

## Introduction

Bromodomain and extraterminal domain (BET) proteins are a family of transcriptional mediators, which assist in the recruitment of RNA Polymerase II (RNA pol II) to enhancers and promoters^1,2^. This family of proteins consists of BRD2, BRD3, BRD4, and BRDT. These proteins bind histone acetylated lysine residues via two highly conserved amino-terminal bromodomains^3^. BRD4 has been extensively studied for its role in transcriptional initiation and elongation^1,2,4–6^. BRD4 interacts with both the mediator complex, and the positive elongation factor B (P-TEFb) at enhancers and promoter regions, respectively^1,7^. BRD4 is expressed in almost all human tissues, and its role in transcription has made it a primary target for possible cancer therapies^8,9^.

JQ1 is a bromodomain inhibitor, which selectively binds to the amino-terminal twin bromodomains of BET proteins^10^. JQ1 treatment displaces BRD4, inhibiting its ability to read acetylated lysine residues^11^. As a result, JQ1 selectively represses the *MYC* oncogene^12^ in a variety of cancer cell lines and animal models of cancer, including acute myeloid leukemia^9^, Burkitt’s lymphoma^13^, and multiple myeloma^12^. JQ1 represses *MYC* expression by interrupting the Mediator-BRD4 complexes located in its super-enhancer region^11,14^. A super-enhancer is a cluster of enhancers within close proximity that are densely populated by transcription factors, active histone marks, and co-activators^15,16^. Super-enhancers are thought to regulate genes that encode proteins that define cell identity as well as proteins that contribute to human disease, including cancers and inflammatory disease^17,18^. In fact, BET inhibitors, such as JQ1 show efficacy in pre-clinical models of cancer as well as autoimmune disease^9,12,13,19–22^.

Despite its potential as a cancer treatment, JQ1 inhibitors repress the expression of multiple genes, not only oncogenes^23^. For example, JQ1 treatment abrogates expression of *IFNG* by memory T-cells^24^. Interferon gamma (IFN-γ) is a cytokine that plays a critical role in both innate and adaptive immunity against viral and bacterial infections. IFN-γ is expressed by effector CD4+ (TH1) and CD8+ (TC1) T cells, memory CD4+ and CD8+ T cells, as well as natural killer (NK) cells and natural killer T (NKT) cells^25–27^. Another BET inhibitor, I-BET 762, was found to repress IFN-γ expression by TH1 cells during development^20^.

Although BET inhibitors have shown efficacy in a variety of pre-clinical models of malignancy, we do not have a complete understanding of its impact on immune cells, nor how long any immunosuppressive effects that exist may last. Here, we sought to evaluate the ability of JQ1 to inhibit production of IFN-γ by TH1 polarized PBMC cultures, CD4+ memory T cells, and NK cells. Our results demonstrate that JQ1 significantly reduces IFN-γ expression in all 3 cells types up to 5 days following treatment. JQ1 does not alter levels of activating H3K27 acetylation (H3K27ac) chromatin marks at the *IFNG* gene locus but displaces RNA pol II from the *IFNG* locus. Finally, inhibition of IFN-γ expression by JQ1 is not irreversible as ability of TH1 polarized PBMC cultures to produce IFN-γ is recovered after removal of JQ1.

## Results

### JQ1 represses *IFNG* expression by TH1 cells, memory T cells and NK cells

**Figure 1:**
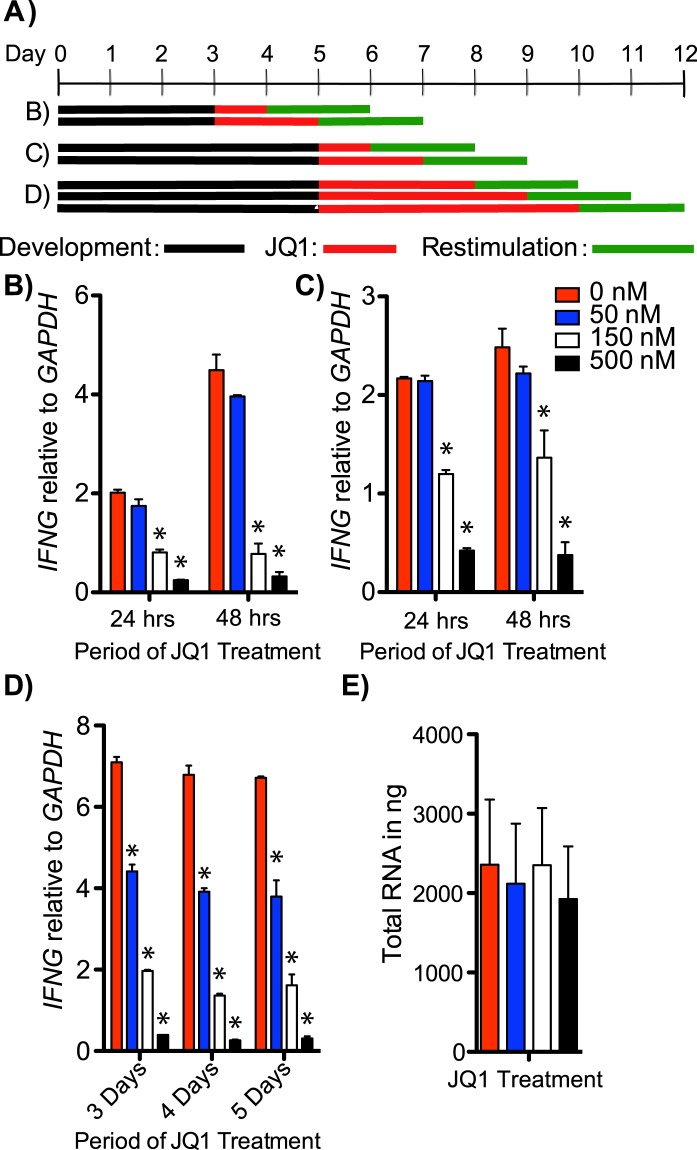
*IFNG* RNA transcripts are significantly reduced in TH1 polarized cultures by the BET inhibitor JQ1. (**A**) Experimental design; black line: period of stimulation with anti-CD3, anti-CD28, IL-12, red line: period of JQ1 treatment, green line: period of restimulation with anti-CD3. (**B**–**D**) Y-axes are levels of *IFNG* mRNA relative to *GAPDH* mRNA, X-axes are treatment times with JQ1, N = 4 each. (**E**) Average total RNA isolated from (**B**,**C** and **D**) cultures at each concentration of JQ1, N = 12. *P < 0.05.

To determine the impact of JQ1 on *IFNG* expression by TH1 polarized PBMC cultures, we treated cells at multiple time points of cell culture. PBMCs were stimulated under TH1 polarizing conditions and treated with 50, 150, and 500 nM final concentrations of JQ1 at different times during the polarization process, harvested and restimulated with anti-CD3 (Fig. 1A). *IFNG* transcripts were significantly reduced in cells treated for 24 or 48 hours with either 150 or 500 nM final concentrations of JQ1 (Fig. 1B). *IFNG* mRNA was also reduced in PBMC treated under TH1 polarizing conditions for 4 or 5 days prior to JQ1 treatment (Fig. 1C). Further, we increased the duration of JQ1 treatment to 3, 4, and 5 days to see if cells would recover *IFNG* expression (Fig. 1D). In each of these treatments, *IFNG* was significantly decreased at all concentrations of JQ1 treatment. Total RNA isolated from cells in culture did not change according to the JQ1 concentration indicating that JQ1 treatment did not have a significant impact on total levels of cellular RNA in the different cultures (Fig. 1E). These results indicate that JQ1 treatment significantly inhibited *IFNG* mRNA expression by TH1 polarized PBMC cultures.

**Figure 2:**
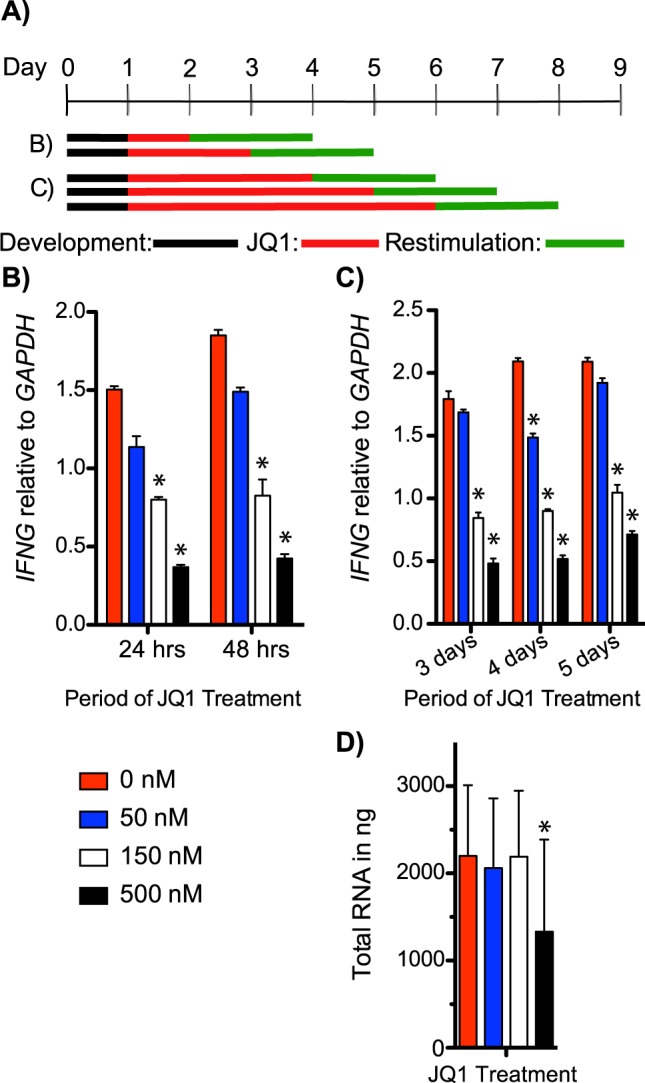
*IFNG* mRNA expression is significantly reduced in CD4+ T memory cells following JQ1 treatment. (**A**) Experiment design; black line: period of stimulation with anti-CD3, red line: period of JQ1 treatment, green line: period of restimulation with anti-CD3. (**B**,**C**) Y-axes are levels of *IFNG* mRNA relative to *GAPDH* mRNA, X-axes are treatment times with JQ1, N = 4. (**D**) Average total RNA isolated from (**B**,**C**) samples at each concentration of JQ1 treatment N = 6. *P < 0.05.

We previously demonstrated that acute exposure of CD4+ memory T cells to JQ1 prevented induction of *IFNG* in response to anti-CD3 stimulation^24^. To expand upon these studies, we stimulated CD4+ T memory cells for 24 hours with anti-CD3 to induce *IFNG* expression, treated cells with varying amounts of JQ1 for varying periods of time, and then re-stimulated cultures with anti-CD3 (Fig. 2A). *IFNG* mRNA expression in memory cells treated with JQ1 for 24 and 48 hours was significantly reduced at 150 and 500 nM concentrations of JQ1 (Fig. 2B). Similarly, when treated for 3, 4, or 5 days, *IFNG* mRNA expression was significantly reduced in CD4+ memory T cells (Fig. 2C). Total RNA isolated from memory cell cultures was significantly reduced in longer term cultures at 500 nM concentrations, which could indicate an impact on cell viability or total RNA expression or both (Fig. 2D). Despite this, *IFNG* mRNA expression was significantly reduced at 150 nM concentrations of JQ1 in CD4+ memory T cells and we found no significant loss of total RNA yield in these cultures. These data indicate that JQ1 treatment reduces *IFNG* mRNA in CD4+ memory T cells, similar to TH1 polarized PBMC cultures.

**Figure 3:**
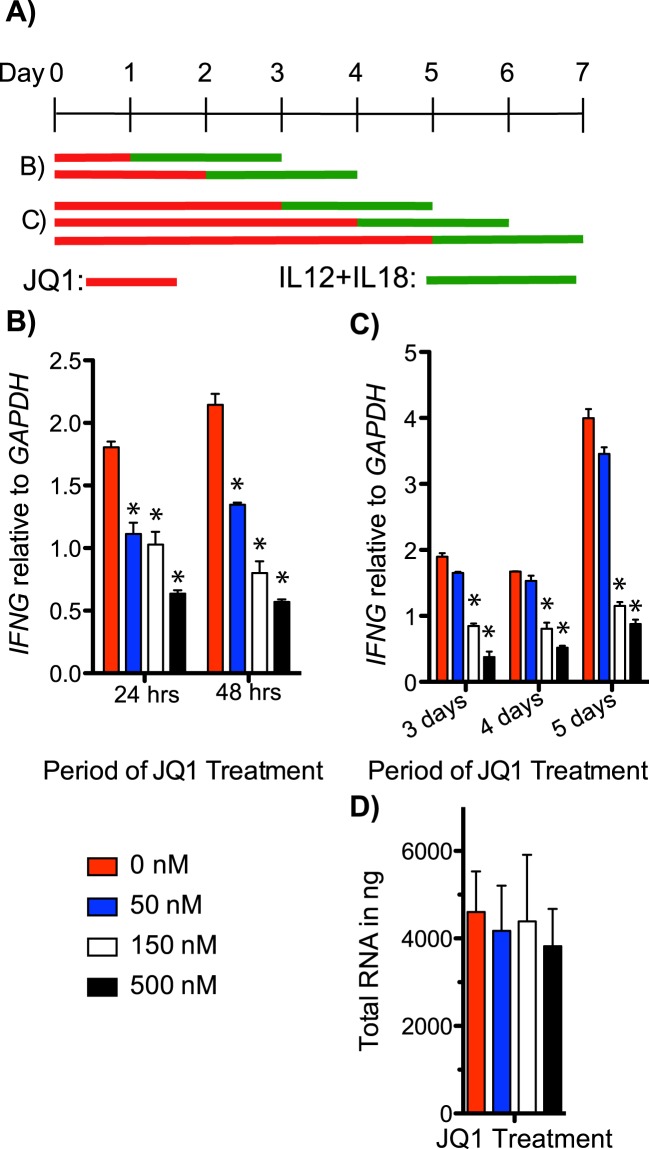
Induction of NK cell *IFNG* mRNA expression is reduced after JQ1 treatment. (**A**) Experimental design; red line: period of JQ1 treatment, green line: period of stimulation with IL-12 and IL-18. (**B**,**C**) Y-axes are levels of *IFNG* mRNA relative to *GAPDH* mRNA, X-axes are treatment times with JQ1, N = 4. (**D**) Average total RNA isolated from samples from (**B** and **C**) at each concentration of JQ1, N = 8. *P < 0.05

We next evaluated effects of JQ1 treatment on NK cells. NK cells were treated with JQ1 for varying periods of time at 50, 150, and 500 nM final concentration and stimulated with IL-12 and IL-18 (Fig. 3A). Similar to TH1 cells, *IFNG* expression was significantly reduced in NK cells treated with JQ1 (Fig. 3B). *IFNG* mRNA was similarly reduced when treated for 3–5 days at 150 and 500 nM concentrations of JQ1 (Fig. 3C). Total RNA isolated from NK cells did not significantly change according to JQ1 treatment, indicating cell viability and total cellular RNA yield were not affected by the JQ1 treatments (Fig. 3D). These results indicate that *IFNG* expression was significantly reduced in NK cells following JQ1 treatment, similar to TH1 polarized PBMC cultures and memory CD4+ T cells.

### JQ1 effects on cell viability

**Figure 4:**
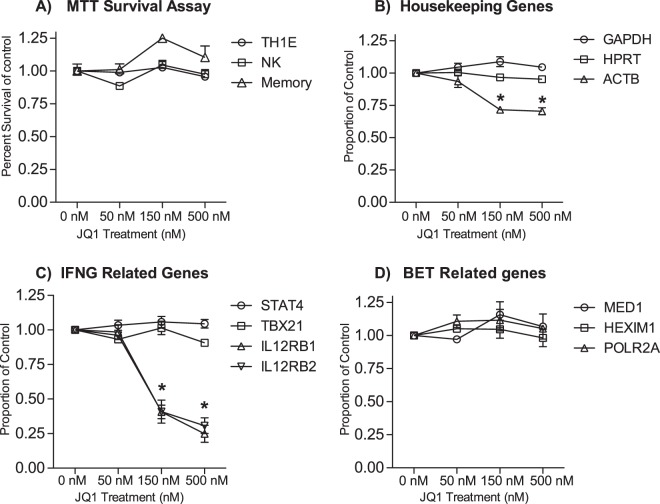
JQ1 treatment is not cytotoxic to TH1 polarized PBMC cultures, but does repress other genes besides *IFNG*. (**A**). Cell cultures were treated with the indicated concentrations of JQ1. Viability was determined after 48 hours using the MTT assay. Results are expressed as percent of the no treatment control, N = 4 (**B**) TH1 polarized PBMC cultures were treated with JQ1 for 48 hour JQ1. RNA was isolated, and analyzed by qPCR. Reactions were standardized to 2 ng/μL of cDNA and calculated relative to 0 nM control. N = 3 (**C**,**D**) As in (**B**), but qPCR results were calculated relative to GAPDH.

We used the ‘MTT assay’ to determine if culture with JQ1 affected viability of the different cell types. We found no loss of viability in TH1 polarized PBMC, NK, or CD4+ memory T cell cultures after treatment with concentrations of JQ1 that significantly diminished *IFNG* expression (Fig. 4A). As a second control experiment, we determined if culture with JQ1 affected expression levels of standard ‘housekeeping’ genes, *GAPDH*, *HPRT*, and *ACTB*. We found that culture with JQ1 did not affect expression levels of *GAPDH* and *HPRT* but reduced levels of *ACTB* by ~25% in TH1 polarized PBMC cultures (Fig. 4B). We also evaluated effects of culture with JQ1 on other genes that encode proteins critical for differentiation and function of TH1, NK, and CD4+ memory T cells, *STAT4*, *TBX21* (T-bet), *IL12RB1* and *IL12RB2*^28^. We found that culture with JQ1 did not affect expression of *STAT4* and *TBX21* but did cause a reduction of *IL12RB1* and *IL12RB2* expression levels (Fig. 4C). Inhibition of expression of *IL12RB1* and I*L12RB2* by JQ1 was similar in magnitude to inhibition of expression of *IFNG*, but these genes are similarly regulated by a super-enhancer^29^. We also examined expression of genes that encode proteins participating in the biologic activity of bromodomain-containing proteins, including *MED1*, part of the mediator complex, *HEXIM1*, part of the *P-TEFb* complex, and *POLR2A*, part of the RNA polymerase 2 complex^30^. We found that culture with JQ1 did not alter expression levels of these genes (Fig. 4D). Thus, under conditions where culture with JQ1 resulted in a marked reduction in IFNG expression levels, changes in viability, expression of ‘housekeeping’ genes, of *STAT4* and *TBX21*, and of *MED1*, *HEXIM1* and *POLR2A* were not observed. However, genes that encode the IL-12 receptor beta subunits were equally sensitive to culture with JQ1 as was *IFNG*.

### JQ1 abrogates RNA Pol II binding to the *IFNG* locus

**Figure 5:**
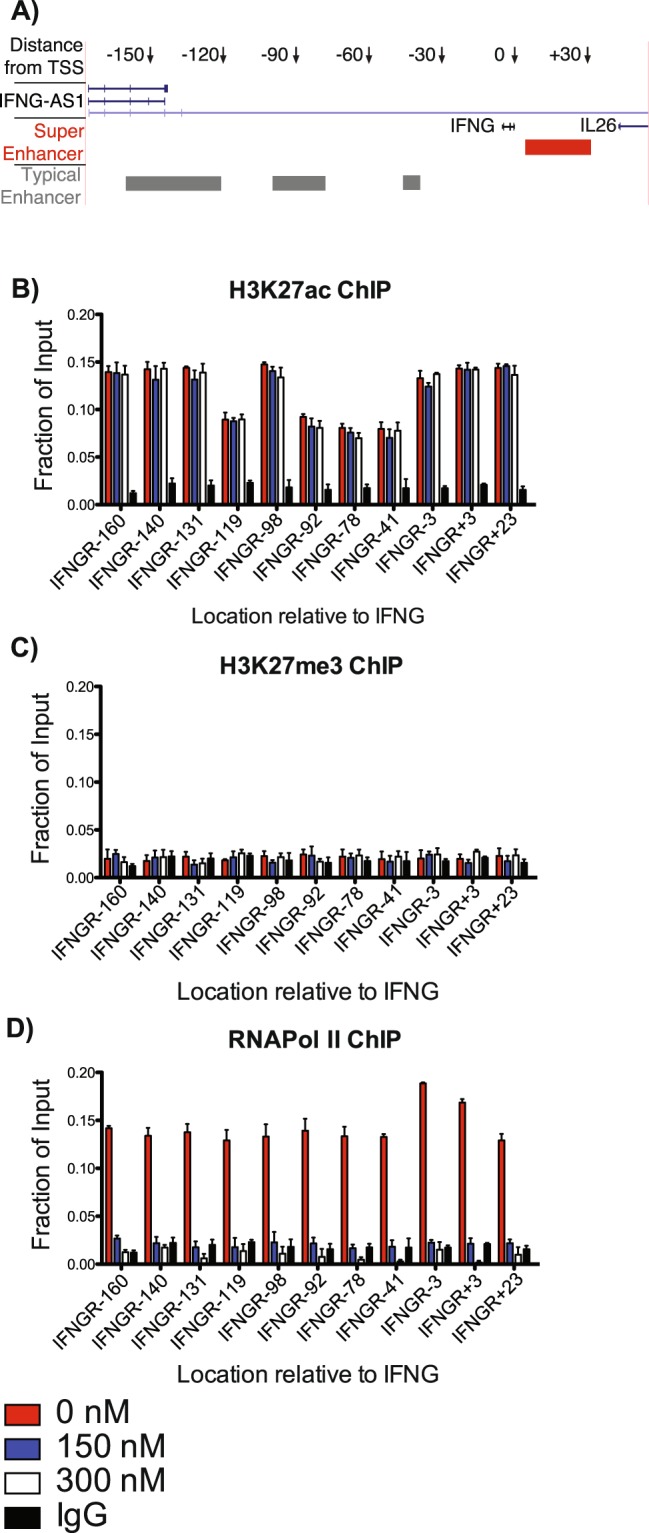
JQ1 treatment does not alter H3K27ac or H3K27me marks but abrogates RNA pol2 binding at the *IFNG* locus. (**A**) Schematic of predicted enhancer locations around *IFNG* locus. Numbers and arrows represent points distance in Kb from transcription start site of *IFNG* gene. Predicted super-enhancers, red line, and typical enhancers, grey line, according to^18,24,29,31^. (**B**) TH1 cells were cultured as in Fig. 1C. ChIP-qPCR assays were performed to measure H3K27ac levels at the *IFNG* locus. Positions, X-axis, are relative to the *IFNG* transcription start site (e.g., IFNGR-160 = 160Kb downstream of TSS), Y-axis is fraction of input DNA, N = 3. Each region evaluated for H3K27ac was significantly higher than IgG control, but did not vary according to JQ1 concentration. (**C**) as in A, but ChIP-qPCR assays were performed to measure H3K27me levels, N = 3. No H3K9me3 ChIP result was significantly different from the IgG control. (**D**) as in A, but ChIP-qPCR assays were performed to measure RNA pol II recruitment, N = 3. RNA pol II ChIP 0 nM controls were significantly different from IgG controls at each location. Similarly, RNA pol II ChIP 0 nM controls were significantly different from JQ1 treatments at every location.

We next sought to investigate epigenetic changes throughout the *IFNG* locus and how chromatin marks may be modified by JQ1 treatment. The *IFNG* gene locus has a large network of enhancers similar to a super-enhancer (Fig. 5A)^18,24,29,31^. These regions are marked by H3K27ac, which make the region more accessible to binding transcription factors and Pol II^15,32^. We cultivated TH1 polarizing PBMC cultures for 5 days, treated with 150 and 300 nM final concentrations JQ1 for 24 hours and isolated chromatin for ChIP assays. We evaluated regions of the IFNG locus previously shown to be highly enriched for H3K27ac marks and recruitment of RNA Pol II^24^. We found that JQ1 treatment did not significantly change the levels of H3K27ac marks throughout the IFNG locus (Fig. 5B). We also analyzed H3K27me3 marks, indicators of an inactive enhancer^33^, and found that chromatin within the *IFNG* locus showed no increase in repressive H3K27me3 marks following JQ1 treatment (Fig. 5C). We similarly performed ChIP assays for RNA Pol II throughout the *IFNG* locus. JQ1 treatment caused a significant decrease in the binding of RNA Pol II both upstream and downstream of the *IFNG* gene (Fig. 5D). Therefore, JQ1 effectively displaced bound RNA Pol II from the *IFNG* locus, but did not change levels of either H3K27ac or H3K27me epigenetic marks at the *IFNG* locus.

### TH1 polarized cultures recover their ability to produce IFN-g after removing JQ1

**Figure 6:**
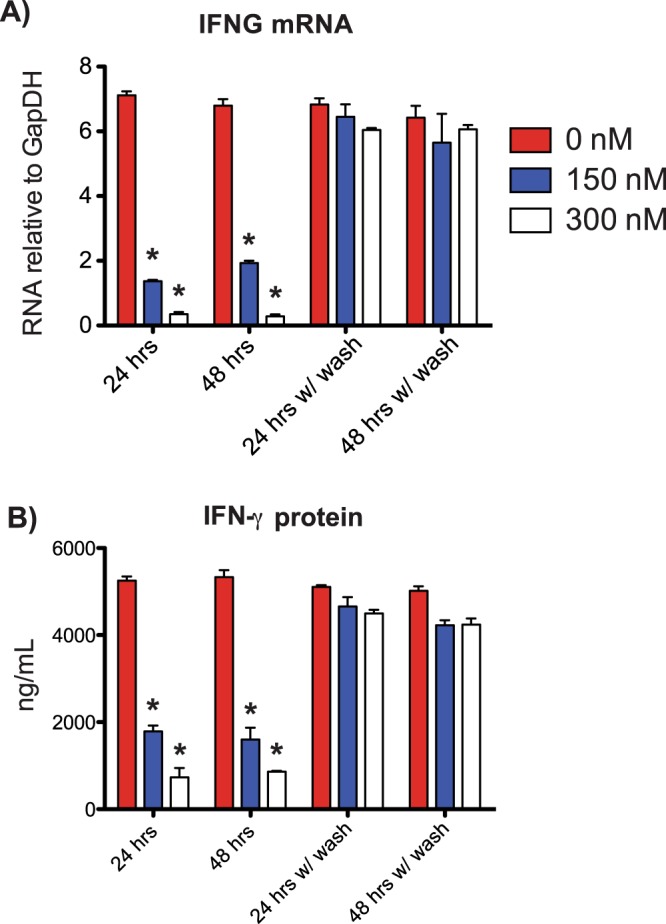
*IFNG* expression recovers following JQ1 removal. (**A**). After 5 days. TH1 cultures were treated with JQ1 at 150 and 300 nM final concentrations for 24 or 48 hours. Cells were either restimulated with anti-CD3 for 48 hours, or washed with new media lacking JQ1 and restimulated with anti-CD3 for 48 hours. RNA was isolated and *IFNG* analyzed by qPCR and normalized to *GAPDH*, N = 3. (**B**) As in A except culture fluids were harvested and IFN-γ levels determined by ELISA, N = 3.

JQ1’s half-life is only 0.9 hours after intravenous injection, or 1.4 hours when administered orally^22^. However, the half-life in tissue culture is not well understood, and we wanted to determine if cells could recover their functions when JQ1 was removed from culture. We treated TH1 polarized PBMC cultures for 24 or 48 hours with JQ1 on day 5 of development, similar to Fig. 1C. Following treatment, cells were either washed and plated with fresh media lacking JQ1 or cultures were continued in the presence of JQ1. We found that *IFNG* mRNA transcripts recovered to pre-treatment levels in TH1 polarized cultures after being washed and re-plated in fresh media (Fig. 6A). We completed a similar experiment but analyzed IFN-γ protein by ELISA. Similarly, IFN-γ was reduced in cultures treated with JQ1 (Fig. 6B), but IFN-γ production also recovered following a wash and re-plating with fresh media, similar to the mRNA results. These results indicate that *IFNG* mRNA and protein levels are reduced following JQ1 treatment but recover to pre-treatment levels following removal of JQ1.

## Discussion

At nanomolar concentrations, the BET inhibitor, JQ1, inhibits expression of *IFNG* mRNA and IFN-γ protein by TH1 polarized PBMC cultures, memory CD4+ T cells, and NK cells. Under these conditions, JQ1 does not interfere with presence of extensive activating H3K27ac marks across the *IFNG* locus nor does JQ1 induce formation of repressive H3K27me marks across the locus. Rather, JQ1 treatment results in almost complete loss of RNA Pol II recruitment across the *IFNG* locus. Further, effects of JQ1 are reversible and removal of JQ1 by media replacement results in complete recovery of *IFNG* mRNA and IFN-γ protein expression by effector TH1 cells. Our results are consistent with a model whereby JQ1 inhibition of *IFNG* expression by TH1 polarized PBMC cultures, memory CD4+ T cells and NK cells results from almost complete loss of RNA Pol II recruitment across the *IFNG* locus. Further, removal of JQ1 allows BET proteins to rebind to the locus and re-establish RNA Pol II recruitment across the *IFNG* locus resulting in efficient *IFNG* expression.

BET inhibitors disrupt function of both typical-enhancers and super-enhancers^14,34–36^. The general view is that functions of super-enhancers and genes driven by super-enhancers are more sensitive to effects of BET inhibitors than typical enhancers^14^. The *IFNG* locus is composed of two large enhancers, each spanning >30 kb, and these have been designated super-enhancers in different studies^18,29^. Almost complete inhibition of *IFNG* expression is achieved at nanomolar concentrations of JQ1. *MYC* and downstream c-*MYC* functions and expression, which require function of a nearby super-enhancer, are also inhibited at similar nanomolar concentrations of JQ1^13,14,22^. Thus, *IFNG* most likely also falls into the class of genes requiring super-enhancers for their expression that also exhibit high sensitivity to BET inhibitors, such as JQ1.

IFN-γ plays a critical role in the adaptive immune response to control infection by intracellular pathogens, including bacteria and viruses, during both initial effector responses and memory responses to infection, as well as malignant transformation and growth^25,37–40^. Major sources of IFN-γ include NK/NKT cells and T cells. When NK/NKT cells immigrate to the periphery, activating epigenetic markings at the *IFNG* locus already exist and these cells are fully capable of producing IFN-γ in response a variety of extracellular stimuli^37,41,42^. In contrast, once in the periphery, naïve T cells have to endure additional developmental programs to produce the required activating epigenetic markings at the *IFNG* locus to allow efficient IFN-γ production in response to stimulation by antigen^39,43,44^. Thus, it might be expected that treatment with BET inhibitors, such as JQ1, *in vivo*, may significantly impair both innate and adaptive arms of immunity that play critical roles controlling infection by intracellular pathogens.

BET inhibitors function by displacing BET proteins from acetylated lysine motifs, but do not directly reverse the chromatin marks^12,45,46^. The repression of *IFNG* in TH1 polarizing PBMC cultures match this model of regulation, as indicated by a continued presence of H3K27ac marks, lack of formation of repressive H3K27me3 marks and displacement of RNAPol II from the IFNG locus following JQ1 treatment. However, *IFNG* expression recovered after removing JQ1 from the cultures at both concentrations. These results indicate that immunosuppressive effects of BET inhibitors, like JQ1, may be reversible.

Certain BET inhibitors have shown very good efficacy in various pre-clinical models of cancer and inflammatory disease^9,12,13,19–22^. It seems likely that BET inhibitors will therefore move forward to actual human clinical studies to treat various malignancies as well as inflammatory diseases. Our results suggest that BET inhibitors may significantly impair both innate and adaptive arms of the immune response, but these effects are reversible. The repression of *IFNG* by JQ1 treatment is observed in the major *IFNG* producing cell types. It remains to be determined if inhibition of the immune response by BET inhibitors will limit their therapeutic usefulness.

## Methods

### Cell isolations and culture

#### TH1 Polarized PBMC Cultures

Total Human PBMCs were isolated from healthy control subjects with no chronic or acute conditions using Ficoll-Hypaque centrifugation. All subjects included in the study were of Caucasian descent between ages 25–32. PBMCs (10^6^ cells/ml) were stimulated with plate bound anti-CD3 (OKT3,CRL-8001, American Type Tissue Collection, ATCC), soluble mouse anti-human CD28 (1 μg/ml; 555725; BD Biosciences) and IL-12 (10 ng/ ml, BD Biosciences) without addition of IL-2 or anti-cytokine neutralizing antibodies essentially as previously described^47,48^. PBMCs were cultured in RPMI 1640 media (11875093, ThermoFisher) supplemented with 10% fetal bovine serum, penicillin-streptomycin and L-glutamine at 37 °C in 5% CO_2_ in air. As outlined in Fig. 1A, cells were treated with JQ1 for varied periods of time, followed by a re-stimulation with anti-CD3 for 48 hours.

#### CD4+ Memory T Cells

Single cell suspensions were prepared from human spleen. CD4+ memory T cells were purified by negative selection (Stemcell, 19157). CD4+ memory cells (10^6^ cells/ml) were stimulated with anti-CD3 for 24 hours as described in Fig. 2A. Cells were treated with JQ1 for varied periods of time, and re-stimulated with fresh plate bound anti-CD3 for 48 hours.

#### Natural Killer cells

NK cells were activated and expanded from human PBMCs using the NK cell activation and expansion kit (Miltenyi Biotec, 130-094-483) for up to a period of 21 days. NK cells were plated in 3 mL cultures at 10^6^ cells/ml, and treated with JQ1 as described in Fig. 3A. After treatment with JQ1, NK cells were stimulated with IL-12 (10 ng/mL: 554613, BD Pharmingen) and IL-18 (10 ng/mL: 4179-25, Biovision) for 48 hours.

JQ1 (SML1524-5MG, Sigma Aldrich) was dissolved in DMSO at a final concentration of 10 mM and diluted into complete medium for addition to cell cultures. The study was approved by the institutional review board at Vanderbilt University Medical Center. Written informed consent was obtained at the time of blood sample collection. Spleen cells were obtained from Tennessee Donor Services under approved protocols with informed consent. All experimental procedures and methods were performed in accordance with relevant institutional guidelines and regulations.

### Quantitative Real-Time PCR

Total RNA isolation, cDNA synthesis using poly-A selection and analysis by qPCR were performed essentially as previously described^42^. All expression levels were normalized to GAPDH using the formula 2^(GAPDH Ct-target gene Ct)^. Primer pairs used in analysis are provided in supplemental Table 1. Housekeeping genes were evaluated by a different calculation in Fig. 4B, to evaluate reference gene quality, using the formula (1/target gene Ct)/(1/target gene Ct at 0 nM treatment). These assays specifically were normalized to total cDNA concentration of 2 ng/μL.

### MTT Cell Proliferation Assay

MTT assays were performed using the CellTiter 96 Non-Radioactive Cell Proliferation Assay (G4001, Promega). Absorbances were determined using an EMax plus Microplate Reader at 570 nm. Cell survival was calculated by (absorbance of treatment/absorbance at 0 nM).

### Chromatin Immunoprecipitation (ChIP)

ChIP procedures were as previously described using anti-H3K27ac (ab4729, Abcam) anti-H3K27me (AB6002, Abcam), anti-RNA Polymerase II (AB817, Abcam), or anti-mouse IgG (SC2357, Santa Cruz) antibodies^44^. DNA was isolated using Pierce Protein A/G magnetic beads (88802, ThermoFisher) via phenol chloroform extraction. Isolated chromatin was analyzed using SYBR-Green qPCR (Applied Biosystems).

### Enzyme Linked Immunosorbent Assay (ELISA)

ELISA was performed according to instructions provided by the manufacturer to analyze IFN-γ protein (BD Bioscience, 555142).

### Statistics

JQ1 treatments and the corresponding qPCR or ELISA analyses were evaluated using a 1 way ANOVA test with Dunnett’s Multiple comparison test for each concentration comparison. ChIP analyses were expressed as fraction of input, and evaluated using an unpaired t-test with Welch’s correction. Unless otherwise stated, *P < 0.05 and data are represented as mean ± S.D.

## Supplementary information

Supplementary Table 1

## Data Availability

No datasets were generated or analyzed during the current study.
