# Supplementary material for: Bromodomain inhibitor JQ1 reversibly blocks IFN-γ production
Source: Sci Rep. 2019 Jul 16;9:10280. doi: 10.1038/s41598-019-46516-x (PMC6635431; doi:10.1038/s41598-019-46516-x)
Supplement: Supplementary file 1 — Supplementary Table 1 [file 41598_2019_46516_MOESM1_ESM.docx]

Bromodomain inhibitor JQ1 reversibly blocks IFN-γ production^1^

Hunter R. Gibbons*, Deborah J. Mi^†^, Virginia M. Farley^†^, Tashawna Esmond^†^, Mary B. Kaood^†^, and Thomas M. Aune^†^*^2^

*Department of Pathology, Microbiology, and Immunology, Vanderbilt University, Nashville, TN 37232

^†^Department of Medicine, Vanderbilt University Medical Center, Nashville TN, 37232

^1^Supported by grants from the National institutes of Health, R01AI044942, R21AI128281, and 5P60DK020593

^2^Address correspondence and reprint requests to Thomas M. Aune, Vanderbilt University Medical Center, MCN T-3113, 1161 21^st^ Ave. S., Nashville, TN 37232. Phone: 615 343-7353, fax: 615 322-6248, e-mail: [tom.aune@vumc.org](mailto:tom.aune@vumc.org)

Supplementary Table 1: Primer sequences used in this study

Target Gene Forward Primer Reverse Primer

IFNG CAGCATCTGACTCCTTTTTCG ATGTCCAACGCAAAGCAATAC

GAPDH AGCCACATCGCTCAGACAC GCCCAATACGACCAAATCC

HPRT CCCTGGCGTCGTGATTAGTG CGAGCAAGACGTTCAGTCCT

ACTB GAGCACAGAGCCTCGCCTTT TCATCATCCATGGTGAGCTGG

STAT4 TGGGACCTGTGCTGAGAGAG GAAGCTGCCTCCCAGTCTTG

TBX21 ACCACCTGTTGTGGTCCAAG GAATGGGAACATCCGCCGTC

IL12RB1 TGTGCGTTCCCCCTGAAAAC ATAGGGCATCTTCCCCAGGT

IL12RB2 CGACACGTGGAAGAATACGGA AGTCACATCGCCTCTCTTGC

MED1 TGTGCGTCAAGTCATGGAGA TGAGATGAGAGCCCAGTCCA

HEXIM1 TTTATTGGGGTGCTCCGCTT TGCAATCTGGGGAGCTCAAG

POLR2A GAGGGAACCTGATGGGCAAA GCCAACCTGGTCAATGGAGA

IFNGR-160 TGCCAGAAAGAAAAGAAGCAG AATTGGCTCACAATTGACCAC

IFNGR-140 AGCACCTGGAGAAGAAGGAAG GCCTGCATTGTCAGCTAAAAG

IFNGR-131 ATCATTTTCCAAGGAGCTGGT TGCTCTGGCATGAGTAGGAAT

IFNGR-119 ATAGCAGGTGAATGCATTTGG ACATATCACATTGGCCCTGAG

IFNGR-98 GCAGTTGTGTGGTGTTGAGTG AGACTCCTCAGCAAATGCAAA

IFNGR-92 CTCTGGAGAAGCCCAAGAAGT TCATCTCTCATCTCCCCACAC

IFNGR-78 AGTTGTTTGGATTGGCTGTTG TTCCATACCCAACAATGTGGT

IFNGR-41 TTGGCCTCAGTTGTATTACGG AAATAGACCTCTGCCCTCCTG

IFNGR-3 GGCACCACTGGATTAGAAACA TGCCTCTCACTCCTCTCATGT

IFNGR+3 CCCGCAATTTGACTCATAAAA TGAACCACTCCTCAAAAATGG

IFNGR+23 CACTTTTCACCCACCTTTGAA ATACCAGATGGGGAAACATCC
